# Challenges and opportunities for modeling coupled human and natural systems

**DOI:** 10.1093/nsr/nwad054

**Published:** 2023-03-07

**Authors:** Yan Li, Shan Sang, Safa Mote, Jorge Rivas, Eugenia Kalnay

**Affiliations:** State Key Laboratory of Earth Surface Processes and Resources Ecology, Beijing Normal University, China; Institute of Land Surface System and Sustainable Development, Faculty of Geographical Science, Beijing Normal University, China; State Key Laboratory of Earth Surface Processes and Resources Ecology, Beijing Normal University, China; Institute of Land Surface System and Sustainable Development, Faculty of Geographical Science, Beijing Normal University, China; Department of Atmospheric and Oceanic Science, University of Maryland, USA; Institute for Physical Science and Technology, University of Maryland, USA; Department of Atmospheric and Oceanic Science, University of Maryland, USA; Institute for Physical Science and Technology, University of Maryland, USA

## Abstract

With the growing recognition of coupled human and natural systems (CHANS), modeling CHANS with two-way feedbacks has become a frontier research area and a critical tool to achieve sustainability. The challenges in CHANS modeling and opportunities to advance its science and application to promote the sustainability of CHANS are discussed in this paper.

The Earth is a very large and complex system that consists of human and natural components interacting bidirectionally with each other, thus forming coupled human and natural systems (CHANS) [1]. The increasing dominance of human impacts, especially after the 1950s [2], marked a new era for the Earth, the Anthropocene, which means humans have become the main drivers of the evolution of changes in the Earth system. Therefore, modeling CHANS is essential for understanding the emergent system properties of CHANS, predicting dynamics and feedbacks (e.g. regime shift, tipping point, and resilience), and informing governance of environmental systems to ensure a path toward sustainability [2,3].

Historically, various modeling methodologies have been developed to model CHANS and their components. These models range from stylized mathematical models [4], system dynamics models [5], and agent-based models [6], to complex integrated assessment models (IAMs) [7] and coupled component models [8] such as the Earth system models [9]. Within these models, some key components of either natural or human systems can be represented with varying degrees of complexity (e.g. physical models such as hydrology, ecosystem, climate, and socioeconomic models of populations, economy, and energy). In contrast, other components are greatly simplified or treated as external drivers.

Currently, the dynamic interactions between natural and human system processes are typically modeled as one-way in existing models, insufficient to represent myriad feedback loops and a wide range of complex behaviors [1]. For example, future climate changes are modeled as the responses of the climate system to prescribed emission scenarios, without considering the feedback of changing climate to human behaviors in society, which in turn impacts emissions [4]. Undoubtedly, these models that use one-way drivers of change significantly advanced our understanding of CHANS. However, only the two-way interactions, which reflect the mutual dependency of the human and natural systems, can capture the actual dynamics of CHANS [2,4]. The absence of these feedbacks may lead to failure to predict the evolution of a coupled system [1].

In this perspective, we discuss major challenges and opportunities for current CHANS modeling and offer suggestions for future directions to advance science and its application to promote the sustainability of CHANS (Fig. 1).

**Figure 1.**
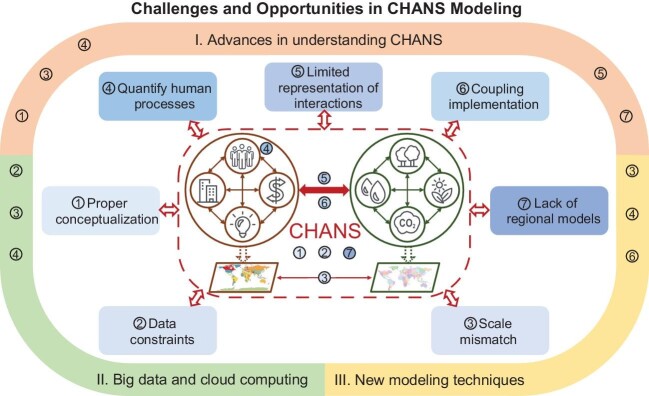
Challenges and opportunities in CHANS modeling. The diagram shows a conceptual graph of CHANS along with seven challenges, including: 1) a proper conceptualization of CHANS, 2) data constraints, 3) scale mismatch, 4) quantifying human processes, 5) limited representation of interactions, 6) coupling implementation, and 7) lacking models at regional scales, as well as three Opportunities (on the outside circle) to help address these challenges, including: I. Advances in understanding CHANS, II. Big data and cloud computing for CHANS, and III. New modeling techniques for CHANS. The Challenge numbers located in the outside Opportunity Circle denote the connection with each specific challenge. [The source figure file is provided in the supplementary files].

**A proper conceptualization of CHANS.** CHANS are heterogenous, with substantially different compositions, structures, processes, and interactions across different scales (e.g. a village, community, city, river basin, region/country, and even the Earth). CHANS modeling requires conceptualizing these systems to a level of complexity that best fits both the research and practical needs. Therefore, a proper conceptualization of the system—including its boundary, structure, and balanced representations of natural and human components, functions, and feedbacks—is a crucial prerequisite for CHANS modeling [3,10]. However, due to the heterogeneity of different CHANS, a universal modeling framework applicable to all cases is yet to be formulated. In practice, CHANS are conceptualized and modeled on a case-by-case basis whose design is guided by prior knowledge with context-specific features [6]. This limits the generalization and transferability of CHANS models from one application/region to another.

**Data constraints.** The modeling of CHANS requires extensive data covering both natural and human science domains. Compared to relatively abundant data sources for the physical environment (e.g. ground monitoring, experiments, field survey, and remote sensing), data availability for human social processes is more limited (especially for demographic and economic sectors), and data collection is more difficult. The common data sources of the human system, such as administrative records and statistical economic data, suffer from inconsistency, coarser resolution, and quality issues, especially for under-developed regions; sociometric survey data at the

individual level are prone to cognitive and sampling biases. Although there have been efforts, such as data access consolidations, by different agencies [10] and a trend towards open data, how to overcome the data constraint and make the best use of data from various sources is still challenging.

**Scale mismatch.** Natural and human processes operate at different spatial and temporal scales, inevitably introducing scale mismatch issues when integrating them together. For example, hydrological processes such as water movement are modeled at sub-hourly (seconds to minutes) and grid/catchment levels. In contrast, socioeconomic processes such as population dynamics, economic growth, and resource demand are modeled yearly at administrative levels (state, country). Coupling these human and natural processes requires novel methodologies to reconcile the scale differences beyond upscaling and downscaling and capture cross-scale dynamics [3].

**Quantifying human processes.** Unlike most natural processes that are based on universal physical laws, human decision making is affected by motivation, emotion, values, culture, policies, and other factors. Human processes, including socioeconomic and social-cultural dimensions, reflect the emergent behavior of individuals’ interactions with the social and natural environments. There are no quantitatively robust theories and principles to predict such processes or solve the adaptive decision problem for social systems. For example, economic models built upon neoclassical economic theory with strong assumptions (e.g. rationality, substitutability) have limited predictability. Due to the lack of process-level scientific explanation, models have to substantially simplify or resort to parameterizations to approximate human processes (e.g. social learning [6]) and their responses to environmental changes (e.g. damage functions based on empirical relationships). However, the estimation of parameter values, especially for those that lack direct measurement, contains large uncertainties. Inadequate quantification of human processes, their responses to natural changes, and uncertainty becomes a major obstacle for endogenously incorporating human systems within CHANS modeling.

**Limited representation of interactions.** The coupling of natural and human systems relies on information exchange between the two systems and their components, which sets the basis for modeling interactions. The common data transfers from human systems to Earth systems include carbon emission, land use change, water demand, GDP, and resource use, while from Earth systems to human systems include climate, ecosystem productivity/services, and crop production [11]. Nonetheless, only a subset of them is included in existing CHANS models. The extent and depth of information exchange between human and natural subsystems are pretty limited, presenting only a weak coupling that is inadequate to capture the complex feedbacks in CHANS. Therefore, improving the representation of CHANS interactions toward deeper integration and an expanded scope by incorporating more transferred variables and their cross-scale feedback networks is the future direction toward strong coupling.

**Coupling implementation.** The technical implementation of coupling in CHANS models requires a properly designed coupling framework to connect various components and enable their communication, especially for coupled component models [8,9]. The choice of coupling frameworks (e.g. loose coupling by file/data exchange, tight coupling by programing all components within the model, or a dedicated coupler to handle the passing of data, parameters, and the scheduling of processes between components) affects the achievable coupling strength and frequency among model components and therefore determines what two-way feedbacks can be implemented [12]. Moreover, the coupling implementation also faces technical challenges (e.g. interoperability barriers [10]) that may require additional modeling efforts, such as necessary model modifications [9] or developing interface/surrogate models [8], depending on the model's complexity. The exact coupling implementation should consider the tradeoffs between complexity, functionality, computation efficiency, and uncertainty while ensuring and maximizing openness, performance, modularity, and consistency of the coupled model. Importantly, interdisciplinary knowledge and a complete understanding of models are critical when coupling models from different disciplines.

**Lacking models at regional scales.** Most CHANS models currently available are established on the global scale [11], targeting large-scale human and natural dynamic issues such as climate change impacts. However, frequent environmental-human conflicts are occurring locally and regionally, while CHANS models built at these scales, which are more relevant for policy and management, are scarce. Regional models focus more on regional processes and pressing issues that global models poorly represent or cannot resolve. For example, how to achieve sustainable crop production in an aquifer relying on groundwater resources, what are the interactions of water, ecology and socioeconomy in a river basin and what impacts do policy interventions have on water security under climate and socioeconomic changes [8]? Therefore, the development of regional CHANS models should be prioritized to facilitate the decision-making process for promoting regional sustainable development. Different roadmaps for regional CHANS models, such as regionalizing global models or building new ones, are viable options. New models are desirable as they have more flexibility in modeling workflow [10,12] (e.g. redesign the system and adopt novel techniques), which can better focus on regional CHANS issues and eventually build up regional integrated model systems and platforms [8].

As a frontier and interdisciplinary research area for the Earth, geographical, and sustainability sciences, new opportunities are present for promoting CHANS modeling.

**Advances in understanding CHANS.** The increasing research efforts on CHANS at multiple scales [11], including theoretical, empirical, and modeling studies, enhance understanding of CHANS components, system behaviors, and feedback mechanisms. The advances in relevant domains of CHANS and increasing interdisciplinary research collaboration lay a holistic system thinking and solid knowledge foundation for CHANS modeling. In particular, various modeling attempts and applications in solving real-world CHANS issues provide valuable lessons to help address the challenges in theory, methodology, and practice of CHANS modeling [3].

**Big data and cloud computing for CHANS.** The emerging big data (e.g. observed, simulated data for natural systems and social sensing [13], and crowd-sourcing data [3] for human systems) provide unprecedented opportunities to address the data constraints for CHANS modeling. Cloud-based services (e.g. Google Earth Engine and various digital data repositories) greatly enhance the capacity for collecting and processing data. Big data, combined with data assimilation [1], benefit process quantification and reduce uncertainty in parameter estimation. More importantly, the rich information of big data allows for discovering unknown patterns and knowledge with the help of deep learning, supporting the data-driven theory and model-building complementary to theory-driven modeling paradigms [14].

**New modeling techniques for CHANS.** New modeling techniques and approaches are developing quickly and offer new insights for CHANS modeling, including machine learning, artificial intelligence, emulator, surrogate models, and model ensembles [14]. The novel methodologies from other fields (e.g. statistical physics, computational social science [13]) and the hybrid use of different techniques (e.g. ABM with reinforcement learning [15], physical models with deep learning [14]) open new possibilities to better characterize the complex human-nature processes and their previously intractable interactions.

Finally, given the complexity and heterogeneity of CHANS, a single universal model might not be feasible. Instead, CHANS models will exist in a hierarchical form of model families. They may consist of different modeling methodologies and strategies (new model, coupling existing models, or hybrid) [11], designed with different levels of complexity and purposes (simple or complex, specific or comprehensive) [3], spanning multiple scales (local, regional, and global scales) and even establishing cross-scale integrated model platforms [8]. These CHANS models should be either science-oriented to better understand and predict the system dynamics, or application-oriented to better manage the system through alternative development pathways. They jointly contribute to formulating the common modeling frameworks/standards and practices much needed for CHANS modeling [10,12]. The emphasized two-way interaction is aspirational for future CHANS model development; admittedly, it is still at an early stage, facing various theoretical and technical challenges (e.g. uncertainty, high sensitivity, and error propagation), and should be approached step-by-step through reciprocal stages of model development and application [3,12]. It also presents a paradigm shift in viewing the rapidly evolving human/nature relationship, now founded equally on a deep understanding of both human and physical systems, as intrinsic parts of a vast coupled system. Therefore, we call for continued research efforts to enrich the theory and methodology of CHANS modeling and release the potential of it to become a major tool to study and improve the sustainability of CHANS.
